# An acute urinary retention in an old man caused by a giant müllerian duct cyst: a case report

**DOI:** 10.1186/1757-1626-2-203

**Published:** 2009-11-18

**Authors:** Mehdi Jaidane, Adnen Hidoussi, Adel Slama, Wissem Hmida, Nabil Ben Sorba, Faouzi Mosbah

**Affiliations:** 1 Urology Department, Sahloul University Hospital, Sousse, Tunisia

## Abstract

Müllerian duct cysts result from an abnormality in regression of the Müllerian system. They may occasionally give rise to symptoms. We report an unusual case of acute urinary retention in an old man caused by a giant Müllerian duct cyst.

A 77-year-old man presented with of acute urinary retention. After bladder drainage, digital rectal examination found a large soft supraprostatic mass. Transrectal ultrasound and computed tomography scans revealed a large multilocular retrovesical cystic mass. The patient underwent open surgical resection of the cyst. Histologically, the cystic lesion was lined with stratified cubocolumnar cells, consistent with a Müllerian duct cyst.

Acute urinary retention in the elderly is not always related to prostatic diseases. Other causes, even congenital ones, may be involved

## Introduction

Müllerian duct cysts result from an abnormality in regression of the Müllerian system [1]. This congenital anomaly is usually diagnosed in pediatric population [1,2] or in adults [3]. Acute urinary retention is not uncommon in the elderly and is mainly caused by prostatic diseases. We report here an unusual case of acute urinary retention in an old man caused by a giant Müllerian duct cyst.

## Case presentation

A 77-year-old Tunisian man of North African origin was admitted to our hospital because of acute urinary retention. He has a 5-month history of urinary frequency and dysuria. Initial physical examination revealed a distended urinary bladder from which 600 ml of clear urine was obtained by catheterization. Digital rectal examination revealed an enlarged, nontender, non-nodular prostate and a large soft supraprostatic mass in the midline. Transrectal ultrasound scan (TRUS) showed a huge retrovesical cyst. TRUS guided needle aspiration of the cyst obtained clear yellowish fluid. The cyst fluid contained no sperm, no malignant cells and was sterile on culture.

**Figure 1:**
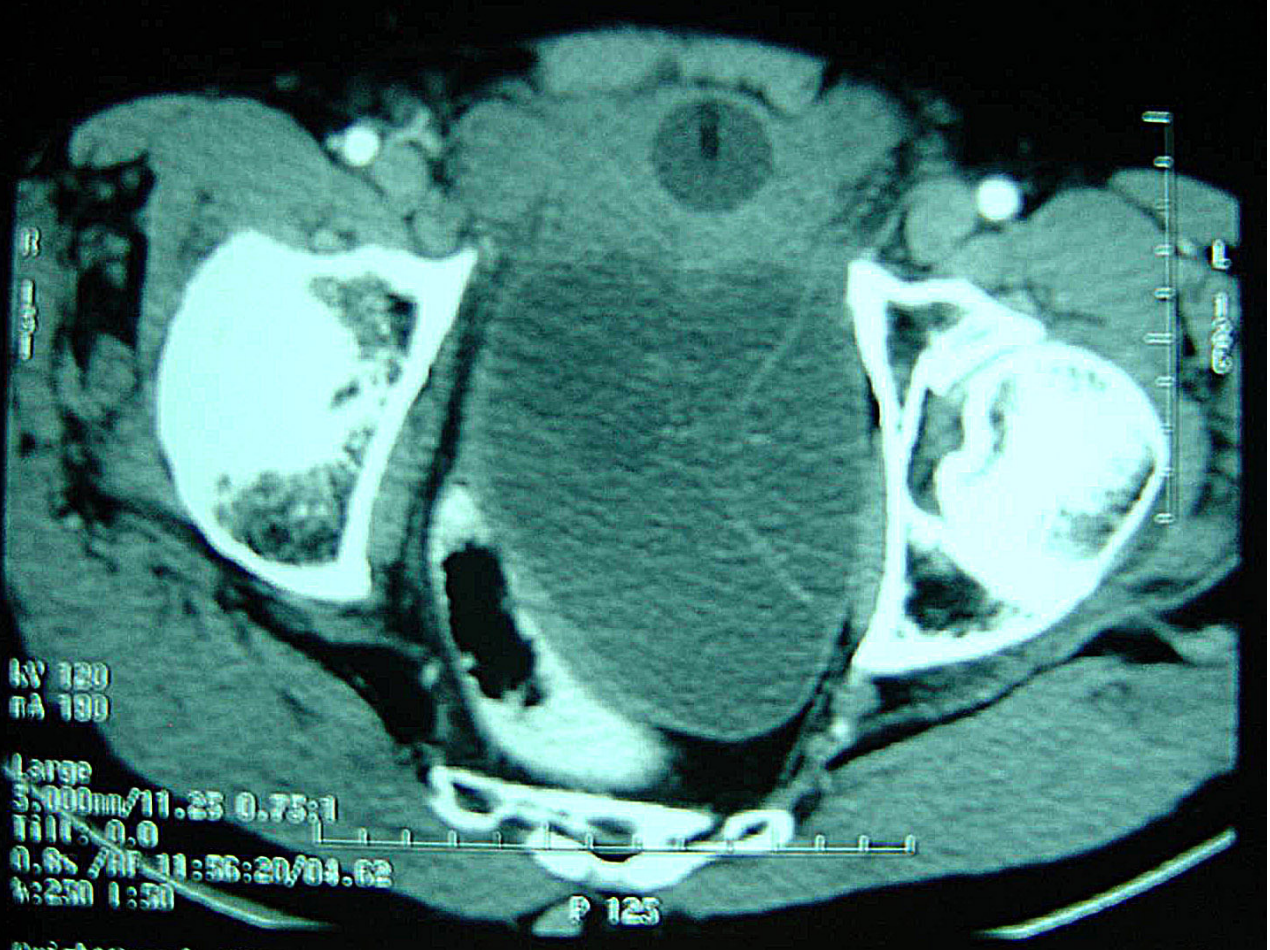
**Computed tomography scan: large multilocular retrovesical cystic mass in the pelvis and abdomen**.

Computed tomography (CT) revealed a large multilocular retrovesical cystic mass in the pelvis and abdomen, with no enhancement after administration of intravenous contrast medium (Figure 1). The cystic mass originated from the midline of the prostate gland, separate from the seminal vesicles, in keeping with a Müllerian duct cyst. There were no other anomalies of the urinary tract.

**Figure 2:**
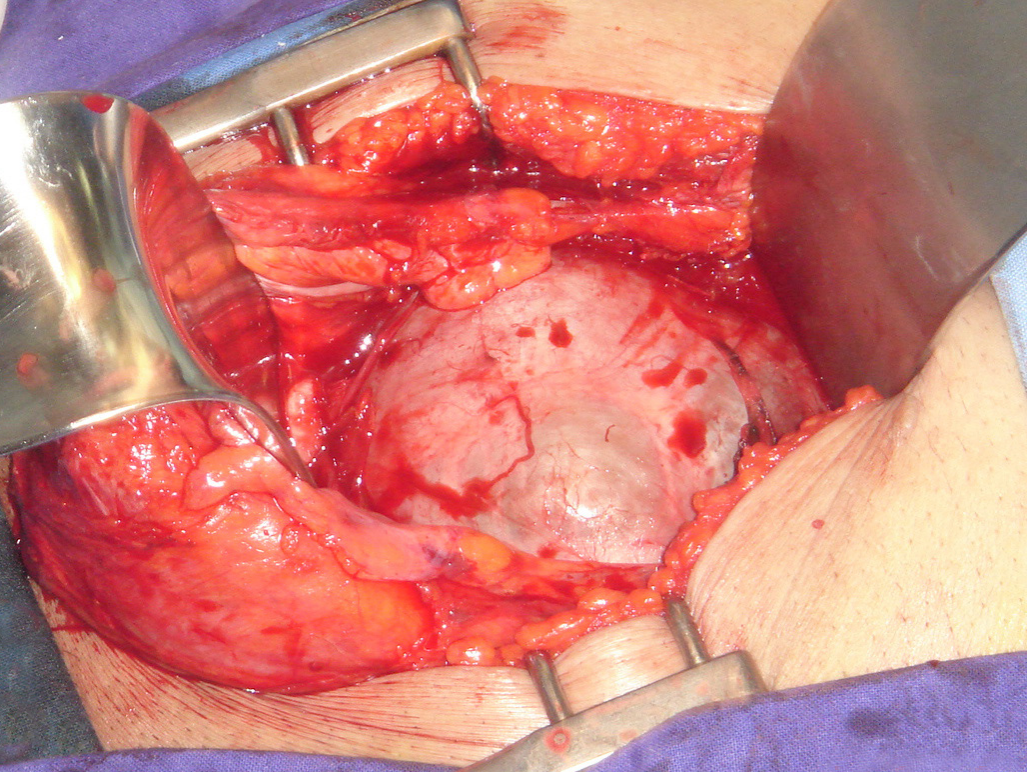
**Intraoperative view: enormous cyst arising from the retrovesical space and extending into the abdomen**.

**Figure 3:**
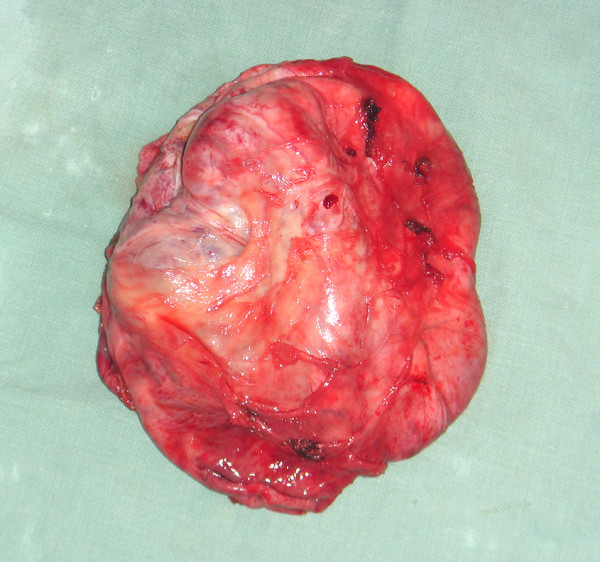
**Gross surgical specimen**.

Surgical exploration was done through a midline infraumbilical incision with a transperitoneal approach. Intraoperatively, there was an enormous cyst arising from the retrovesical space (Figure 2). The cyst was mobile and not significantly adherent to the bowel or pelvic side wall. With further dissection the cyst was believed to be clearly extra peritoneal in origin with a neck at the base of the prostate. Removal of the cyst in this area necessitated removal of a limited cuff of prostatic tissue. Peroperative urethrocystoscopy exclude communication of the cyst with urethra. The resected cyst was a multilocular cyst weighing filled with 2000 ml of clear yellowish fluid (Figure 3).

Postoperative course was uneventful and the patient regains normal voiding function. Histologically, the cystic lesion was lined with stratified cubocolumnar cells, consistent with a Müllerian duct cyst.

## Discussion

Müllerian duct remnants, which include Müllerian duct cysts and enlarged prostatic utricles, are midline prostatic cystic lesions [1]. In the male, the müllerian ducts regress under the effect of Müllerian inhibiting factor produced by the Sertoli cells of the testes at about the 10th week of fetal life [1]. Müllerian duct remnants result from incomplete Müllerian duct regression. English is generally credited with the first description of a Mullerian duct remnant in 1873 [4]. There is some confusion in the literature as the terms dilated utricle and Mullerian duct cyst tend to be used interchangeably [4,5]. Although these entities share a similar embryologic origin, they are clinically different [1,3]. Müllerian duct cysts tend to develop between the 3rd and 4th decades of life. The external genitourinary system is usually normal. Reported prevalence in older autopsy series in men is 1% [6]. They may be underreported as some authors found a prevalence of 5% in urologic patients [3]. These cysts are typically round and do not communicate with the urethra [1,3]. They are commonly characterized by small retroperitoneal extensions in the midline. Variants occupying the entire pelvic region or extending into the abdomen, as in our patient, are a rare clinical entity [6].

Prostatic utricle cysts represent a distinct entity that results from dilatation of the prostatic utricle. These midline masses are typically smaller and communicate with the posterior urethra. They develop in the first or second decade of life and are associated with various genitourinary abnormalities, including hypospadias [1,3].

Most Müllerian duct cysts are asymptomatic but they may present with irritative urinary symptoms (urinary frequency, urgency), obstructive symptoms (dysuria, decreased urinary flow rate), hematuria, hematospermia, bloody urethral discharge, ejaculatory pain, urinary tract infection, epidydimitis, infertility or constipation [1,3,7]. Malignant degeneration is a rare complication [6].

Acute urinary retention is also a clinical presentation described in children [8] or adults [9]. In literature review, we found only one single previously reported case of acute urinary retention due to Müllerian duct cyst in an old man [10].

Learning from this new case report is that acute urinary retention in the elderly is not always related to prostatic diseases. Other causes, even congenital ones, may be involved.

Transrectal ultrasound, CT, and magnetic resonance scanning are the most useful diagnostic tools [3,7,9]. Different treatment options are available for Müllerian duct cysts. Transurethral resection and percutaneous aspiration are performed for small Müllerian duct cysts [9]. Laparoscopic excision has been reported [11]. In large pelvic or abdominal cyst, open surgical excision is the treatment of choice [1,7].

Müllerian cysts are not so rare and they are probably underreported [3]. As they are asymptomatic in most cases, treatment is indicated only in symptomatic cases [3].

## Consent

Written informed consent was obtained from the patient for publication of this case report and accompanying images. A copy of the written consent is available for review by the Editor-in-Chief of this journal.

## Competing interests

The authors declare that they have no competing interests.

## Authors' contributions

MJ and AH collected the data and literature review, and wrote the manuscript. AS and WH revised and provided comments on the manuscript. NBS and FM were the attending doctors, carried out the surgical procedure and literature review. All authors read and approved the final manuscript.
